# Assessing the Costs and Cost-Effectiveness of Genomic Sequencing

**DOI:** 10.3390/jpm5040470

**Published:** 2015-12-10

**Authors:** Kurt D. Christensen, Dmitry Dukhovny, Uwe Siebert, Robert C. Green

**Affiliations:** 1 Department of Medicine, Brigham and Women’s Hospital, Harvard Medical School, Boston, MA 02115, USA; 2 Department of Pediatrics, Oregon Health and Science University, Portland, OR 97239, USA; E-Mail: dukhovny@ohsu.edu; 3 Department of Public Health, Medical Decision Making and Health Technology Assessment, University for Health Sciences, Medical Informatics and Technology, Hall in Tirol 6060, Austria; E-Mail: uwe.siebert@umit.at; 4 Department of Health Policy and Management, Harvard School of Public Health, Boston, MA 02115, USA; 5 Department of Radiology, Massachusetts General Hospital, Harvard Medical School, Boston, MA 02114, USA; 6 Department of Medicine, Brigham and Women’s Hospital, Harvard Medical School, Partners Personalized Medicine, Boston, MA 02115, USA; E-Mail: rcgreen@genetics.med.harvard.edu

**Keywords:** whole genomic sequencing, whole exome sequencing, cost, cost-effectiveness

## Abstract

Despite dramatic drops in DNA sequencing costs, concerns are great that the integration of genomic sequencing into clinical settings will drastically increase health care expenditures. This commentary presents an overview of what is known about the costs and cost-effectiveness of genomic sequencing. We discuss the cost of germline genomic sequencing, addressing factors that have facilitated the decrease in sequencing costs to date and anticipating the factors that will drive sequencing costs in the future. We then address the cost-effectiveness of diagnostic and pharmacogenomic applications of genomic sequencing, with an emphasis on the implications for secondary findings disclosure and the integration of genomic sequencing into general patient care. Throughout, we ground the discussion by describing efforts in the MedSeq Project, an ongoing randomized controlled clinical trial, to understand the costs and cost-effectiveness of integrating whole genome sequencing into cardiology and primary care settings.

## 1. Introduction

In 2007, the cost to sequence an entire genome was approximately $10 million [1]. Sequencing costs have fallen fast since then, and the $1000 genome will soon be a reality [2,3,4,5]. Widespread use of whole genome or whole exome sequencing (heretofore, *genomic sequencing*) is quickly becoming feasible. Some experts even believe that genomic sequencing of individuals at birth will become commonplace [6].

Yet, major questions remain about the impact genomic sequencing will have on overall health care expenditures. The growth of health care spending has outpaced inflation in most countries for the last five decades [7], and especially in the United States where health care spending accounts for over 17% of GDP [8]. Many hope that genomic sequencing can help to bend the cost curve by streamlining diagnostic processes [9,10] and by informing selection and dosing of treatments to maximize efficacy, to minimize costly, life-threatening adverse events, and to avoid expensive treatments in patients who are unlikely to benefit from them [9,11]. Identification of pathogenic variants and variants associated with an increased risk for actionable conditions may help providers and patients target prevention efforts, avoiding more expensive procedures associated with treating disease [12,13,14]. Given that the capabilities of genomic sequencing continue to expand, some are optimistic that integrating genomic sequencing more fully into clinical settings may reduce overall health care expenditures [15].

Concerns are also great, however, that regular use of genomic sequencing will substantially increase health care expenditures while providing limited clinical benefits. The majority of physicians, for example, expect genomic testing to increase health care costs in the future [16]. The infrastructure requirements for clinics to use and store genomic sequencing findings can be tremendous [17], and in contrast to the costs of generating DNA sequences, the costs of variant interpretation may not fall quickly in the future [18]. More importantly, genomic sequencing results are often inconclusive and require additional medical follow-up [19,20]. Identification of secondary findings during genomic sequencing may initiate a cascade of confirmatory testing and follow-up screening that may or may not be warranted [21,22,23]. At present, reviews of the cost-effectiveness of disclosing secondary findings from genomic sequencing have shown a tremendous lack of existing evidence [24,25,26]. This lack of data has become a barrier in the willingness of payers to cover genomic sequencing services [27].

Although the utility of sequencing or lack of it will ultimately determine how often it is used in clinical settings, understanding its full costs and cost-effectiveness will be critical as payers make decisions about reimbursement. This commentary provides an overview of the potential economic impact of integrating genomic sequencing into clinical settings, with an emphasis on germline testing and applications in general medicine. We begin with an overview of the cost of genomic sequencing, describing the factors that have driven costs to date and the costs that are likely to be of greatest importance in the future. We then address the utility of diagnostic and pharmacogenomic applications of genomic sequencing, addressing what is also known about the cost-effectiveness. In each section, we ground our discussion by describing methods of an economic evaluation we are conducting alongside the MedSeq Project, an ongoing randomized clinical trial of whole genome sequencing (WGS) in cardiology and primary care settings [28]. The purpose of this commentary is to provide an overview of the costs and cost-effectiveness of genomic sequencing, to identify strategies that will help advance cost-effectiveness research on these topics, and to orient readers about research strategies to provide data about the cost-effectiveness of integrating WGS into medicine.

## 2. The Full Costs of Genomic Sequencing 

The “$1000 genome” has long been a goal of genomic sequencing proponents, a critical cost threshold which would place sequencing in line with other advanced diagnostic tests [1,29]. The field has been rapidly approaching this goal, by any accounting. However, the costs of integrating genomic sequencing into clinical settings are unlikely to fall as rapidly in coming years as the costs of producing raw DNA sequences become comparable to the costs of variant interpretation. Additionally, the infrastructure costs to facilitate clinicians’ use of sequencing are likely to remain substantial, and the costs to follow-up of sequencing results are uncertain. It is useful to deconstruct the steps of genomic sequencing [30] to better understand what the overall costs may be in the future. 

### 2.1. DNA Sequencing

DNA is first collected with a blood draw or tissue sample and extracted. Library generation then occurs, where random DNA fragments are created that contain adapter sequences that are complementary to platform-specific PCR and sequencing primers. DNA is fragmented, and additional processing (e.g., end-repair, A-tailing, “barcoding”) is completed. In most cases, PCR amplification of the library is needed before sequencing.

The library is then sequenced at an internal or external service using a platform suited to the demands of the situation (see Xuan *et al.* for an overview of popular sequencing platforms [31]). To date, this step of generating the DNA sequence has traditionally been the most time-intensive and expensive, constituting the vast majority of calculations of the “cost of DNA sequencing” [32] and associated media reports about the falling costs of DNA sequencing. Improvements to the costs of sequencing are well-documented, and reflect fundamental changes in sequencing strategies as well as technical improvements in computing power. Next generation sequencing platforms that rely on massively parallel sequencing of millions of DNA fragments simultaneously replaced first-generation platforms that used Sanger-based chemistries and capillary-based instruments. This change precipitated a sharp drop in costs around 2008 [32,33]. Strategies that rely on single-molecule sequencing are emerging now, promising to drop the costs of sequencing even further [31].

After curation (see below), laboratories may also need to confirm the analytic validity of variants before reporting findings to ordering physicians. Confirmation is typically completed with an FDA-approved sequencing method, such as Sanger sequencing [34].

### 2.2. Variant Interpretation

Typically, DNA sequencing data is returned to the ordering laboratory in SAM or BAM formats, along with variant call files for further processing. Laboratories process the data to align reads, to determine sequencing depth, and to convert files into formats appropriate for bioinformatics analysis. According to site- and context-specific computer algorithms, variants are filtered according to what’s known about them in public and proprietary databases, their frequency within the families of probands and reference populations, and functional predictions (e.g., exon *vs*. non-coding regions, loss-of-function). Given that public databases often exaggerate the pathogenicity of specific variants [35], a final set of variants is usually curated manually to ensure results are not inaccurately classified. Access to allele frequency databases, such as the Exome Aggregation Consortium Browser (ExAC), and databases of variant interpretations, such as NCBI's ClinVar, will continue to reduce the number of variants per genome that will require manual review. However, given the rate of novel variants that will not be present in curated datasets and the time needed to assess or reassess variants that do meet criteria for review [18], variant interpretation costs are unlikely to decrease at the same rate as the costs of generating DNA sequences.

### 2.3. Medical Care and Follow-Up

Ultimately, the costs outlined above may be relatively insignificant compared to the downstream costs of medical procedures ordered in response to genomic sequencing findings. In diagnostic and treatment-related contexts where patients are already managing symptoms, primary findings are less likely to provoke follow-up procedures that would not have been initiated otherwise. However, most laboratories have adopted American College of Medical Genetics (ACMG) recommendations to proactively query an additional 56 genes for potential disease-causing variants, regardless of why sequencing was initiated [22]. In fact, most sequencing programs have developed proprietary lists to query a greater number of genes for secondary findings [36,37,38]. Although ACMG recommendations have been updated to permit patients to “opt out” of analysis for secondary findings [39], the preponderance of evidence suggests that few patients will choose to do so [40,41,42]. 

It is in the context of secondary findings disclosure and sequencing of well populations that the risks of unnecessary medical follow up may be particularly salient [43]. Variants that are identified as pathogenic by informatics tools are sometimes reclassified as benign following manual curation [21,44]. If laboratories are not careful, medical costs may be accrued not only to treat or screen patients for conditions they would never manifest, but also for side effects that may be associated with those procedures. Furthermore, penetrance estimates for pathogenic variants are often derived from high-risk populations and may not be as strong among well populations [45]. Sequencing may detect true-positive variants that are treated, but would not have become symptomatic during patients’ lifetimes. Combined with some physicians’ tendencies to “do something” in response to information [46], secondary findings disclosure and genomic sequencing of well populations may result in overdiagnosis and overtreatment, and unnecessarily increase costs and harm patients.

Depending on what is being studied, analyses may include additional costs both within and outside of the health care system. These expenditures may include patient co-pays for physician visits, testing or treatments [47], time off from work for health care appointments [48], potential life and disability insurance complications [49], and genetic testing of other family members [24]. What costs to include and omit depends on the perspective of the analysis.

### 2.4. Infrastructure

The majority of the discourse about genomic sequencing costs has focused on the per-patient costs of sequencing, reporting, and medical follow-up. The infrastructure demands of genomic sequencing are also high. Variant interpretation may require facilities, personnel, and software, that are tailored to the needs of immediate analyses [18], the needs for ongoing re-analyses, and integration of genomic information with other types of health information [17]. Data storage, maintenance, transfer, and analysis are also likely to remain considerable, and are expected to constitute a growing percentage of overall sequencing costs in the future [17]. There may even come a time when re-sequencing patients becomes less costly than storing a patient’s file for re-analysis. Additionally, professional groups, expert panels, and physicians themselves recognize a need for educational programs that are specific to the demands of genomic sequencing [50,51,52,53,54]. Addressing these needs is likely to require a substantial investment by health care systems. 

### 2.5. Economic Evaluation alongside the MedSeq Project

Preliminary findings from the MedSeq Project are beginning to provide insight about the magnitude of the costs outlined above. The MedSeq Project is a randomized trial of WGS in clinical care. Study procedures are published in detail elsewhere [28,55]. Briefly, we randomized ostensibly healthy primary care patients and cardiology patients with diagnoses of hypertrophic or dilated cardiomyopathy to review family history information with their participating physician or to review their family history along with WGS findings. WGS reports in the MedSeq Project describe identified monogenic risks for disease, carrier risks, pharmacogenomic findings for 5 common drugs with PharmGKB Clinical Annotation Levels of Evidence Class I and Class II [56]. A cardiac risk supplement also provides genome-based cholesterol level predictions and risk predictions for eight phenotypes associated with cardiovascular disease based on SNP profiling [57]. Patients are followed for six months following review of results, and data is collected from questionnaires, interviews, and medical records reviews to understand the medical, behavioral, and economic impact of WGS.

Alongside the primary clinical trial, we are evaluating the costs of WGS. Costs for garnering informed consent are calculated by recording the length of informed consent sessions and applying wage data for a nurse per the Bureau of Labor Statistics (BLS) [58]. Costs for genomic sequencing include market rates for sequencing at the Illumina Clinical Services Laboratory using the Illumina HiSeq platform [59] and costs from the Laboratory of Molecular Medicine (LMM) to confirm variants via Sanger sequencing. LMM also tracks the amount of time its staff spends at each stage of variant interpretation, and costs are calculated by applying BLS wage rates for a medical scientist and laboratory director to those times, as applicable. Finally, medical care costs for the six months following disclosure of results are calculated by reviewing billing data from the Partners HealthCare Research Patient Data Registry and standardizing costs using reimbursement schedules from Medicare’s Physician Fee Schedule and Outpatient Prospective Payment System [60,61]. Additional information is being collected about patient out-of-pocket expenses, including time off from work, patient copayments, and transportation costs for medical appointments. An overview of how costs are being calculated in the MedSeq Project is presented in Table 1. 

**Table 1:** Strategy for tracking participant-level costs in the MedSeq Project.

Step	Per-Participant Cost Strategy
Informed consent	Length of informed consent X BLS wage data
DNA sequencing	Market costs for WGS on Illumina HiSeq PlatformLMM tracked-costs for Sanger confirmation
Variant interpretation	Tracked time X BLS wage data
Medical care through 6 months	Billing codes per patient records, standardized to CMS reimbursement schedules
Patient out-of-pocket expenses	Adapted from the Medicare Expenditure Panel Survey [62]

## 3. The Utility and Cost-Effectiveness of Genomic Sequencing

There are hopes that the widespread integration of genomic sequencing into medicine, while associated with upfront investments, will reduce downstream health expenditures. However, prevention and treatment programs rarely result in an overall cost savings [63]. The more relevant question is whether genomic sequencing can be cost effective and improve health and wellbeing at a similar or even better value when compared with other well-accepted interventions in health and medicine [64].

Genomic sequencing can provide insight into a vast array of health-related information, from susceptibility to infection [65] to responses to dietary choices [66] to geographic ancestry [67]. Its most common current uses, however, are to identify and diagnose disease, and to inform treatment selections. Here, we provide an overview of what is known about the efficacy and cost-effectiveness of genomic sequencing in diagnostic and pharmacogenomic applications.

### 3.1. Diagnostic Applications

The utility of genomic sequencing for diagnosing disease has been tested in a limited number of contexts [25,26,68,69]. It has had notable successes in pediatric settings. An observational study of 2000 consecutive patients who received whole exome sequencing (WES) through the Whole Genome Laboratory of Baylor College of Medicine (primarily pediatric patients) found that sequencing achieved diagnoses in 25% of cases. Furthermore, 58% of the diagnostic findings had not previously been reported [37]. The diagnostic yield among acutely ill children may be even higher. Using trio and familial approaches, a children’s hospital in the central U.S. attempted to use genomic sequencing to determine the cause of neurodevelopmental disorders among children. WGS and/or WES identified a disease-causing variant in 73% of families with children that had acute neurodevelopmental delays and 40% of families with children that had non-acute neurodevelopmental delays [70]. In a similar retrospective analysis of infants with acute illness, Willig and colleagues noted that neonatal and pediatric intensive care units at Children’s Mercy–Kansas City achieved diagnoses for 57% of patients who received rapid-turnaround WGS compared to 9% of patients who received standard genetic testing [71].

Patients with rare disease also have benefitted greatly from the emergence of genomic sequencing. Of 814 consecutive patients with suspected genetic disorders who received WES through at the University of California, Los Angeles, Clinical Genomics Center, 26% received diagnoses, with a higher success rate using trio-sequencing rather than singleton testing [72]. A national effort in Canada to identify genetic causes of childhood-onset disorders, the Finding of Rare Disease GEnes (FORGE), was able to achieve diagnoses for 29% of 362 families who had attempted and failed to achieve diagnoses previously using WES [73].

Assessments of the diagnostic yield of sequencing in other settings are limited. An international effort used targeted sequencing to achieve diagnoses for 11% of patients with intellectual disabilities who had failed to achieve diagnoses through more traditional methods, including CGH microarray analysis [74]. What was particularly notable about this effort is that it achieved diagnoses using singleton testing rather trio analysis. Furthermore, cancer genetics clinics of the University of Texas Southwestern Medical Center and the Ohio State University cancer genetics programs were able to identify genetic variants associated with cancer predispositions in 21% of 82 non-BRCA cancer patients using WGS, although the study was conducted for research purposes and results were not disclosed to patients [19].

Each of the previously-mentioned studies included sick patients whose condition might be impacted by a diagnosis or explanation for their condition. In the context of secondary findings and genomic sequencing of healthy populations where identified variants may or may not result in future disease, the clinical importance of an identified variant is less clear. However, data about how often secondary findings are identified can provide some information insight about the potential yield we could anticipate from genomic sequencing if it was used for population screening. ClinSeq investigators noted seven deleterious *BRCA1/2* variants and one deleterious *SDHC* variant among 572 participants (~1% of the study population) [38]. Amendola and colleagues analyzed data from the NHLBI Exome Sequencing Project, finding pathogenic or likely pathogenic variants in approximately 2% of the 4,300 European-ancestry participants and 1% of the 2203 African-ancestry participants when examining a panel of 112 genes associated with potentially medically actionable genetic disorders [36]. Analyzing a database that included over 11,000 exomes, Gambin and colleagues found that 5.6% of participants had a variant in one of the 56 ACMG actionable genes that had been reported in ClinVar to be pathogenic [75]. Of note, this study did not manually curate data, which may explain the higher rate of findings they observed.

Health economic evidence on genetic screening is limited, and much work needs to be done to establish the utility of genomic tests before addressing questions about cost-effectiveness [76,77]. Cost-effectiveness has been properly evaluated in genomics for only a few conditions and contexts. Genomic sequencing appears to be cost-effective for diagnosing neurodevelopmental disorders [70]; and evaluations of Lynch syndrome screening approaches suggest that genomic sequencing can be cost-effective, but not currently as a first-line testing strategy [78,79]. A scoping review identified cost-effectiveness studies had been conducted for fewer than one-third of conditions recommended by ACMG for secondary findings screening. Even worse, the cost-effectiveness of screening for these conditions in the general population was examined for only 8% of the conditions [26]. Initial modelling work suggests that secondary findings disclosure is cost-effective in diagnostic contexts, but not cost-effective for general population screening at present [24]. 

### 3.2. Pharmacogenomic Applications

The majority of cost-effectiveness research on genetic testing has been to evaluate potential pharmacogenomic applications [25]. Pharmacogenomic information about drug metabolism can provide invaluable insight about how to dose medications to maximize their efficacy. Furthermore, pharmacogenomic testing can identify patients who may have adverse, life-threatening responses to drugs. Dosing and allergy associations are important enough for some drugs that FDA guidelines for drug labelling may require genetic testing prior to administration. Examples of these medications include carbamazepine, ivacaftor, pimozide, and tetrabenazine [56]. In addition, the FDA requires genotyping of tumors before initiation of numerous cancer treatment drugs, including imatinib and panitumumab [56]. 

Knowledge about pharmacogenomic associations is expanding rapidly. However, these advances have primarily occurred in the context of cancer care, and require tumor analysis rather than germline testing. Table 2 summarizes pharmacogenomic findings for drugs approved by the FDA in another common area of treatment, cardiology care, including cost-effectiveness information per a brief PubMed review of published analyses for each drug. As it shows, the evidence base that genetic information should be an important factor when making drug selection or dosing decisions is limited. For only one medication (clopidogrel) was genetic testing recommended to ensure it would be effective for patients of all genotypes. The table also shows that cost-effectiveness evidence about disclosing pharmgogenomic information to inform cardiology care is lacking overall, and generally corresponds with FDA recommendations.

**Table 2:** FDA-approved drugs with pharmacogenomic information relevant to cardiology care.

Drug	Genes	FDA Recommendation*	Evidence of Cost-Effectiveness
Clopidogrel	CYP2C19	Recommended	Strong [80,81,82,83]
Ticagrelor	CYP2C19	Actionable	Moderate [69,82]
Carvedilol	CYP2D6	Actionable	Not assessed
Propafenone	CYP2D6	Actionable	Not assessed
Warfarin	VKORC1, PROS1, PROCA	Actionable	Weak [84,85]
Warfarin	CYP2C9	Informative	Weak [84,85]
Prasugrel	CYP2C19, CYP2C9, CYP3A5, CYP2B6	Informative	Weak [69,82]
Isosorbide & Hydralazine	NAT1-2	Informative	Not assessed
Metoprolol	CYP2D6	Informative	Not assessed
Propranolol	CYP2D6	Informative	Not assessed
Quinidine	CYP2D6	Informative	Not assessed

* PharmGKB categorizes FDA-approved labels about pharmacogenomic implications into four categories [56]: *required*, where genetic testing should be conducted before drug use; *recommended,* where genetic testing is recommended before drug use; *actionable*, where labels suggest that genetic variation may affect drug efficacy, dosage or toxicity; and *informative*, where labels simply address that genes may be involved in drug metabolism or pharmacodynamics.

### 3.3. Clinical Utility and Cost-Effectiveness in the MedSeq Project

The clinical utility of genomic sequencing is being assessed in the MedSeq Project by recording the number of pathogenic and likely pathogenic monogenic findings for each participant. Whether and how WGS reports impact patient care is also being assessed through self-administered questionnaires that physicians complete after each disclosure session and through end-of-study interviews. One comprehensive method to assess overall patient outcome is to evaluate the health related quality of life differences, using quality-adjusted life years (QALYs). At baseline and 6 months after disclosure of MedSeq Project results, patient participants complete the SF-12, version 2 [86], a validated, commonly used 12-item instrument with well-established reliability and validity that assess health-related quality of life. We will transform SF-12 data into SF-6D values. The SF-6D is a generic preference-based single index measure of health (*i.e.*, a utility) that ranges from 0 to 1 and can be used to generate QALYs [87]. Although a 6-month follow-up period is probably too short to observe an impact on health-related quality of life among a study population that does not have diagnostic or treatment-related reasons for undergoing WGS, the data we are collecting will provide a critical foundation for larger and longer-term studies. 

Cost-effectiveness will be assessed using incremental cost-effectiveness ratios (ICERs) by dividing the differences in costs by the differences in QALYs when comparing both randomized groups. We will also conduct subgroup analyses and secondary analyses to assess whether benefits, harms, and ICERs of WGS differ between by cohort (primary care *versus* cardiology) and to assess the impact of having a monogenic finding disclosed. 

## 4. Discussion

The falling costs of sequencing have facilitated the integration of genomic sequencing into medicine. We can expect costs to continue to fall over the short-term, although the true price of sequencing may plateau soon as steps that cannot be as readily automated—expert curation, clinical reporting, infrastructure maintenance—grow relative to the cost of generating a DNA sequence, and complete automation in variant classification will not occur for some time. More importantly, the impact of genomic sequencing on downstream medical care is currently unknown, and is likely to drive cost-effectiveness assessments in the future, particularly with respect to secondary findings and genomic sequencing of healthy populations. If genomic sequencing in those contexts identifies potential threats early and aids in prevention, it may be prove to be an invaluable, efficient health-promotion resource. If, instead, it motivates confirmatory testing and long-term screening with limited clinical benefits, its use may need to be encouraged more selectively. The balance of these outcomes will determine how expensive, how beneficial (or harmful), and how cost effective genomic sequencing will be. The MedSeq Project is one of a number of ongoing efforts to understand the impact of integrating genomic sequencing into clinical care. The findings from this study and others like it are helping professional organizations and policymaker develop approaches to better ensure the benefits of genomic sequencing are maximized and provide value relative to its high, if falling, cost.

While the purpose of this commentary is to orient researchers to the challenges of assessing the costs and cost-effectiveness of genomic sequencing, a number of key recommendations should be emphasized. First, novel study designs are needed to assess the long-term impact of genomic sequencing, particularly regarding secondary findings. Although randomized controlled trials represent a “gold standard” for assessing the utility of emerging therapies and tools, their time horizons should are often insufficient for capturing all relevant benefits, harms and costs. Decision-analytic modelling and cohort simulations covering sufficiently long time horizons (*i.e.*, usually patients’ lifetimes), can overcome some of these problems [88]; but more creative methodologies utilizing clinical data are also needed, at a minimum, to inform penetrance estimates of monogenic findings that would be disclosed as secondary findings.

Second, analyses of genomic sequencing need to be sensitive to advancements in sequencing approaches. An expanding array of sequencing platforms are becoming available, each with its own strengths and limitations with respect to the costs of generating raw DNA sequences, as well as factors that may affect the utility of sequencing such as speed and accuracy. Furthermore, systems have been developed to integrate genomic sequencing data more fully with other related data, such as gene-expression and phenotypic information. These advances may help laboratories classify variants more efficiently by providing evidence that supports or refutes the pathogenicity of an identified variant, particularly if the variant is related to a secondary finding that is unrelated to the original reasons sequencing was ordered. In addition, the data presented earlier highlights how differences in sequencing approaches can impact its effectiveness. Trio sequencing, in particular, has been more effective than singleton sequencing in achieving diagnoses [73]. As DNA sequencing costs continue to drop, the costs of sequencing trios nears the cost of sequencing an individual, making trio sequencing a more efficient option when feasible. 

Finally, new methods are needed that integrate a wider array of potential benefits, harms and costs from genomic sequencing. Complex multi-disease decision-analytic models are needed to comprehensively research these issues, including conditions where patient out-of-pocket expenses may be particularly high [88]. Models may need to be expanded to consider the implications of identifying germline variants for other family members, too. Moreover, the mechanisms by which genomic sequencing can affect outcomes for a given condition may be myriad. For instance, sequencing may affect pulmonary outcomes by both identifying inherited cardiovascular risks and providing information about appropriate medications. Researchers will need to be creative about their approaches to evaluating genomic sequencing. The literature on lifestyle interventions may provide a starting point, given the multidimensional impact they can have on health and wellbeing [89,90].

## 5. Conclusions

This review focuses on diagnostic and treatment-related applications of germline genomic sequencing, despite its growing utility in other settings. The evidence base is expanding quickly for specialized applications, such as cell-free DNA sequencing for prenatal testing [91,92] and tumor sequencing for oncology care [68,93,94,95,96], not to mention epigenetic, RNA, and microbiome sequencing. Moreover, the utility of genomic sequencing may increase as understandings of gene-environment interactions improve. How the field integrates those approaches into patient care is likely to be predicated on the policies and infrastructure developed for germline genomic sequencing. The urgency to understand the cost-effectiveness of genomic sequencing will only continue to grow as prices continue to drop and new applications emerge.
